# Quantitative *E. coli* Enzyme Detection in Reporter Hydrogel-Coated Paper Using a Smartphone Camera

**DOI:** 10.3390/bios11010025

**Published:** 2021-01-19

**Authors:** Kawaljit Kaur, Winny Chelangat, Sergey I. Druzhinin, Nancy Wangechi Karuri, Mareike Müller, Holger Schönherr

**Affiliations:** 1 Physical Chemistry I & Research Center of Micro and Nanochemistry and Engineering (Cμ), Department of Chemistry and Biology, University of Siegen, Adolf-Reichwein-Straße 2, 57076 Siegen, Germany; Kawaljit.Kaur@uni-siegen.de (K.K.); chelangat.winny16@students.dkut.ac.ke (W.C.); m.mueller@chemie-bio.uni-siegen.de (M.M.); 2 Mechatronics Engineering Department, Dedan Kimathi University of Technology, Nyeri-Mweiga Road, Nyeri 10143, Kenya; 3 Chemical Engineering Department, Dedan Kimathi University of Technology, Nyeri-Mweiga Road, Nyeri 10143, Kenya; nancy.karuri@dkut.ac.ke

**Keywords:** bacteria detection, paper-based biosensors, *Escherichia coli*, β-glucuronidase, reporter hydrogels, lab-in-a-phone

## Abstract

There is a growing demand for rapid and sensitive detection approaches for pathogenic bacteria that can be applied by non-specialists in non-laboratory field settings. Here, the detection of the typical *E. coli* enzyme β-glucuronidase using a chitosan-based sensing hydrogel-coated paper sensor and the detailed analysis of the reaction kinetics, as detected by a smartphone camera, is reported. The chromogenic reporter unit affords an intense blue color in a two-step reaction, which was analyzed using a modified Michaelis–Menten approach. This generalizable approach can be used to determine the limit of detection and comprises an invaluable tool to characterize the performance of lab-in-a-phone type approaches. For the particular system analyzed, the ratio of reaction rate and equilibrium constants of the enzyme–substrate complex are 0.3 and 0.9 pM^−1^h^−1^ for β-glucuronidase in phosphate buffered saline and lysogeny broth, respectively. The minimal degree of substrate conversion for detection of the indigo pigment formed during the reaction is 0.15, while the minimal time required for detection in this particular system is ~2 h at an enzyme concentration of 100 nM. Therefore, this approach is applicable for quantitative lab-in-a-phone based point of care detection systems that are based on enzymatic substrate conversion via bacterial enzymes.

## 1. Introduction

Pathogenic bacteria are a major worldwide concern, e.g., in the areas of food and water safety as well as healthcare. Independent of the local setting and level of development, any shortage or even lack of robust, reliable and affordable tests for rapid detection of pathogenic bacteria leads to a possible elevated transmission risk of pathogens, which may subsequently lead to life-threatening illnesses. This is true for some highly developed countries, where the screening of patients regarding colonialization with antimicrobial resistant bacteria is still not carried out routinely due to cost and resource issues. Likewise, bacterial contaminations in food and drinking water pose serious threats to the well-being of people particularly in less-developed countries. The World Health Organization (WHO) declared that 1.1 billion people lack access to an improved drinking water supply [1]. For people living in regions that have no access to necessary technical and economic resources, microbiological water lab tests in approved laboratories are often practically out of reach.

Independently of the focus and local challenges, the development of novel cheap, rapid, easy to use point of care (POC) bacteria detection methods may contribute to overcome the limitations of conventional methods especially regarding the specific needs for certain application areas and settings. Key to their success are also efficient methods to quantify their performance in order to set proper thresholds for clear readouts.

In this context, there has been growing interest in detection of (pathogenic) bacteria, such as the hygiene marker *Escherichia coli* (*E. coli*) on-site using simple approaches that can be applied by non-specialists in a non-laboratory setting. *E. coli* is by default tested in drinking water or other industrial water resources based on detection of the target enzyme β-Glucuronidase (ß-Gus), an enzyme that is produced in high concentration by 95% of all *E. coli* [2,3] but also by mammalian cells as a lysosomal enzyme responsible for degradation of glucoronate-containing glycosaminoglycans [4] as well as in some plants [5], that as off-target sources obviously do not result in relevant false positives in the β-Gus chromogenic standards method approved for specific *E. coli* detection. Conventional methods for detecting and identifying bacteria are based on culturing the microorganism on chromogenic differential nutrient agar plates. These can be also be tailored for the selective growth of a particular bacterial species and thus can provide useful information about the bacteria present in a contaminated sample [6]. The drawback is that these methods are time-consuming due to the necessary transport to specialized microbiological laboratories in hospitals or research institutes that need to incubate these specific agar plates for at least one up to 4 days for visual detection of bacteria colonies by bare eye. Additionally, there might be challenges with antagonistic organism interference, lack of specificity, or special challenges for slow-growing fastidious microorganisms. Other highly advanced approaches require trained personnel or dedicated and highly complex analytical devices, such as mass spectrometers, flow cytometers that can detect fluorescently labeled individual bacteria in complex media in an automated manner, as well as thermocyclers for polymerase chain reaction (PCR) for detection of short DNA sequences specific for a certain bacterial species [7,8].

Hence it is not surprising that a multitude of approaches with novel sensing materials has been reported in recent years. These approaches rely on, among other things, sensing nanoparticles [9,10], nanocapsules [11,12] and hydrogels [6,13,14,15,16,17,18]. The adaptation of such bacteria detection approaches with demonstrated feasibility to POC-compatible formats for application in remote settings without controlled temperature and reliable electricity is another challenge. Suitable, but yet unavailable sensor systems, must exhibit pronounced robustness, long shelf-life and high stability, coupled with ease of application and a robust and bias-free detection and analysis.

Due to the worldwide availability of mobile phones, smartphone-based biosensors have already been gaining considerable momentum in this area and opened new avenues towards next-generation POC sensing and biosensing applications, also known as “lab-in-a-phone technology” [19,20]. For instance, Kim et al. reported on a smartphone-based sensor for determining blood hematocrit from 10% to 65% with a limit of detection (LOD) of 0.1%. In their study the hematocrit concentration was determined from the red, green and blue components of images captured under white light illumination in a microfluidic device using a smartphone camera [21,22]. Liu et al. [23] proposed a smartphone sensor to measure concentrations of the antibiotic streptomycin in different food products. The detection of changes in absorbance ratio due to streptomycin binding to aptamer-conjugated Au nanoparticles was realized by a smartphone camera [21,23]. Likewise the concentrations of proteins (bovine serum albumin, BSA), enzymes (catalase) and carbohydrates were determined using an analysis of the average brightness of smartphone-acquired images, which were obtained by converting the image pixels to HSV (hue, saturation, value) color space [21,24].

The combination of lab-in-a phone technology with smart interface or nanochemistry technologies for bioanalytical applications has raised hope in particular for applications in remote and resource-poor settings [19,25]. As carriers for the reporting units, paper and paper-based microfluidic devices have been developed for applications in areas such as health diagnostics, food safety, and environmental monitoring [26]. Paper eliminates the need for pumps, since the wicking mechanism allows for passive transport via capillary action. Moreover, paper is also compatible with a variety of chemicals and biomaterials (biological inertness) and eliminates problems associated with bubbles [27,28].

**Figure 1:**
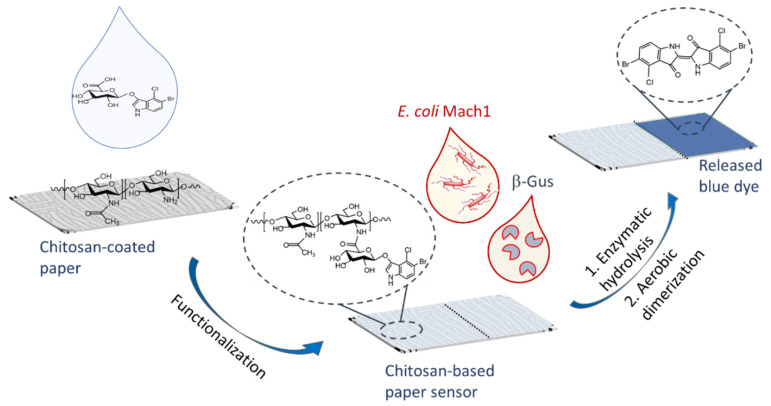
Schematic of paper-based detection of the bacterial enzyme ß-Gus, which is produced by more than 94% of all strains of *E. coli* [30]. An ultrathin coating of the cellulose fibers with functionalized chitosan affords the colorimetric sensing. The enzyme catalyzed reaction of the chromogenic substrate 5-bromo-4-chloro-3-indolyl-β-D-glucuronide (X-Gluc) affords in the presence of oxygen a deep blue indigo derivative, which is water-insoluble and can be quantified by adequate analysis of smartphone camera photographs.

Hence a promising strategy is the combination of feasible bacteria detection approaches with a paper-based carrier system that was investigated here for the *E. coli* marker enzyme β-glucuronidase (ß-Gus) and the non-pathogenic *E. coli* Mach1 lab strain as a model organism (Figure 1) with smartphone-based detection, readout and quantitative data analysis. Previously, chitosan hydrogels equipped with different fluorogenic or chromogenic substrates were shown to rapidly, selectively and sensitively detect and differentiate among different strains of bacteria with LOD for the enzymes in several cases <1 nM after 1 h observation time [16,29]. As we report here, chitosan-based autonomously self-reporting hydrogels were successfully adapted to this paper carrier-based format to detect and quantify concentrations of bacterial enzymes. In particular, the robust and complete analysis of images, captured with a conventional smartphone camera, affords quantitative values for the limit of detection. This new approach that was demonstrated for the detection of *E. coli* can be adapted for use in other sensing systems that rely on color changes.

## 2. Results

The previously reported chitosan sensing hydrogel approach was first adapted to a paper substrate format [16]. The modification of filter paper with chitosan as well as the subsequent functionalization with reporter moieties was thoroughly characterized with surface analytical methods and field-emission scanning electron microscopy (FESEM). Subsequently, the smartphone-based data acquisition and analysis of the captured data using a new and refined analysis was addressed to afford values for the LOD.

### 2.1. Characterization of Chitosan-Modified Paper and Functionalization

Filter paper with pore sizes of 5–12 µm and 12–15 µm was modified with chitosan by repeated casting from 1% acetic acid solution, followed by drying. Depending on the concentration and number of deposition cycles, the static water contact angle of chitosan-coated filter paper approached 112°, while the unmodified paper had a contact angle <10°–15° (Figure S1). After two deposition cycles, the contact angles were consistently higher compared to one deposition. Likewise, for paper with larger pore sizes, higher contact angles were observed.

**Figure 2:**
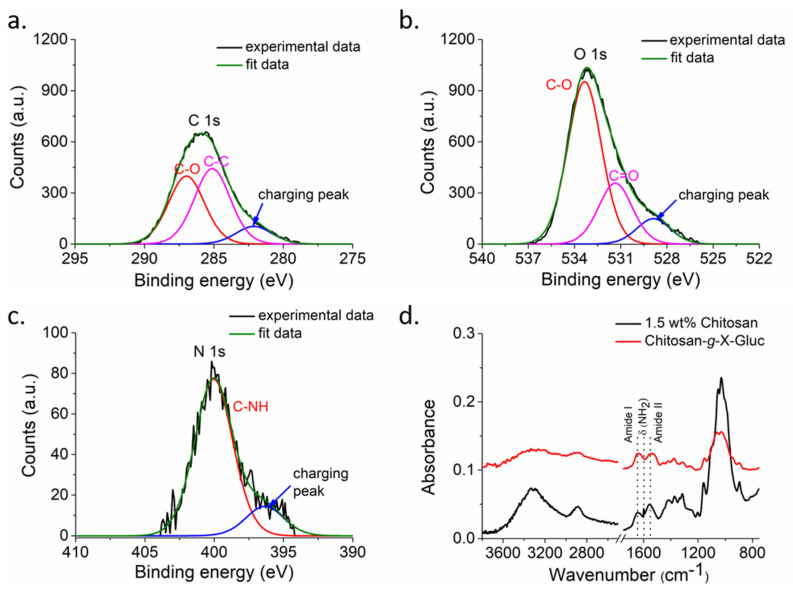
X-ray photoelectron spectroscopy (XPS) high-resolution spectrum of 1.5 wt% chitosan deposited on paper substrate for (**a**) C1s, (**b**) O1s and (**c**) N1s. (**d**) attenuated total internal reflection (ATR)-Fourier transform infrared (FTIR) spectra of paper modified with chitosan (sample coated twice using 1.5 wt% chitosan solution in 1% acetic acid, black trace) and chitosan functionalized with the chromogenic substrate X-Gluc (sample coated twice using 1.5 wt% chitosan solution in 1% acetic acid, red trace).

The presence of the chitosan on top of the filter paper was confirmed by X-ray photoelectron spectroscopy (XPS). In the survey spectra (Figure S2), only C1s and O1s signals were detected for uncoated paper, while a clear N1s signal was observed in addition to the C1s and O1s signals for the chitosan-coated samples (see also Table S1). The detailed XPS element scans acquired for chitosan coated specimens (Figure 2) show the corresponding C1s, O1s and N1s signals. In all spectra a significant contribution of uncompensated charging was observed, which was corrected for by fitting a separated peak. The C1s signal (Figure 2a) was deconvoluted into two peaks at 286.9 and 285.1 eV, which are attributed to carbon species bonded to oxygen (C–O) and aliphatic carbon (C–C), respectively. The oxygen signal (Figure 2b) contained contributions of oxygen as C–O at 533.3 eV and C=O at 531.5 eV. Finally, the corresponding N1s signal was attributed to N in –C–NH, centered at 400 eV (Figure 2c).

The wet chemical functionalization of the chitosan coated paper was carried out using established protocols that rely on the activation of carboxylic acid groups of the chromogenic 5-bromo-4-chloro-3-indolyl-β-D-glucuronide (X-Gluc) by *N*-(3-dimethylaminopropyl)-*N*-ethylcarbodiimide hydrochloride-*N*-hydroxy succinimide (EDC-NHS) chemistry ) followed by conjugation to the primary amino groups of the chitosan coating. The formation of covalent bonds between X-Gluc and chitosan can be concluded from the attenuated total internal reflection (ATR)-Fourier transform infrared (FTIR) spectra acquired prior to and after the reaction (Figure 2d). The disappearance of the deformation vibration centered at 1599 cm^−1^ upon reaction and the altered contributions of amide I and amide II peaks is consistent with the successful attachment of X-Gluc (Table S2).

FESEM analysis unveiled that the chitosan covered the cellulose fibers and formed a comparatively smooth film with some nanoscale porosity on top, which retained its structure also after functionalization with the chromogenic substrate X-Gluc (Figure S3). Closer inspection of the microscopy data shows that for the uncoated filter paper, single fibers could be easily identified. Uncoated paper exhibited a rougher surface, Figure S3a. Chitosan forms a continuous film that covers the paper fibers, as it was observed for 1.5 wt% and 2 wt% chitosan for coating, see Figure S3c,d, respectively. Paper coated with 1 wt% chitosan solution had films that were not continuous, see Figure S3b, suggesting lower amounts of chitosan coating, likely also due to the lower viscosity of the 1 wt% chitosan solution compared to solutions with higher concentrations.

### 2.2. Enzymatic Reactions of Chitosan-Modified Paper Functionalized with Chromogenic Substrate

The X-Gluc functionalized chitosan-coated paper shows in test experiments the development of blue color, consistent with previously reported data (Figure S4). The difference among a film coated once or twice with chitosan is significant: The twice coated and then functionalized sample shows under otherwise identical conditions more intense color.

**Figure 3:**
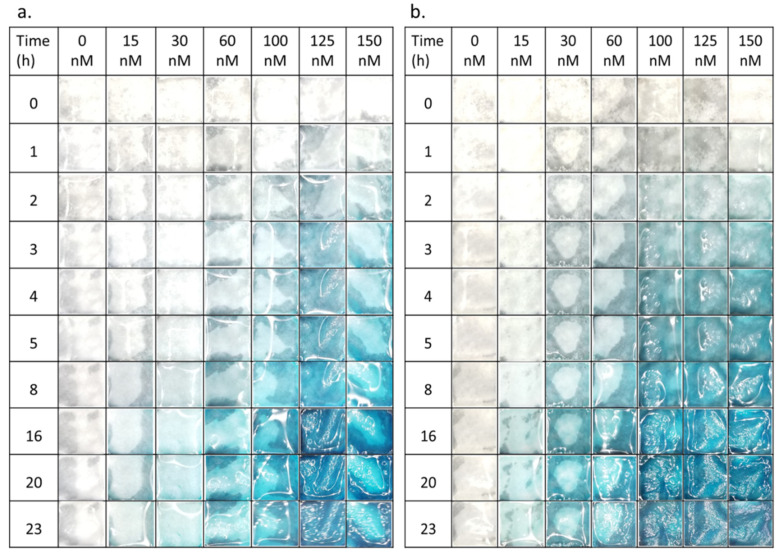
Photographs of visible color change of chitosan-g-X-Gluc coated paper after incubation in different ß-Gus concentrations (**a**) in phosphate-buffered saline (PBS) and (**b**) in lysogeny broth (LB).

For studying the enzymatic reaction systematically, 100 µL of the sterilized ß-Gus in phosphate-buffered saline (PBS, pH 7.4) or in lysogeny broth (LB, pH 7.0) were diluted to a designated concentration of the enzyme and deposited on the functionalized paper in a custom-made sample holder that was placed at 25 °C in a black box. Photographs of the test paper covered with solution were taken at different time intervals with a conventional mobile phone camera under white light illumination (Figure 3). Depending on the concentration and reaction time, the characteristic blue color of the indigo-like dimerized dye was observed.

**Figure 4:**
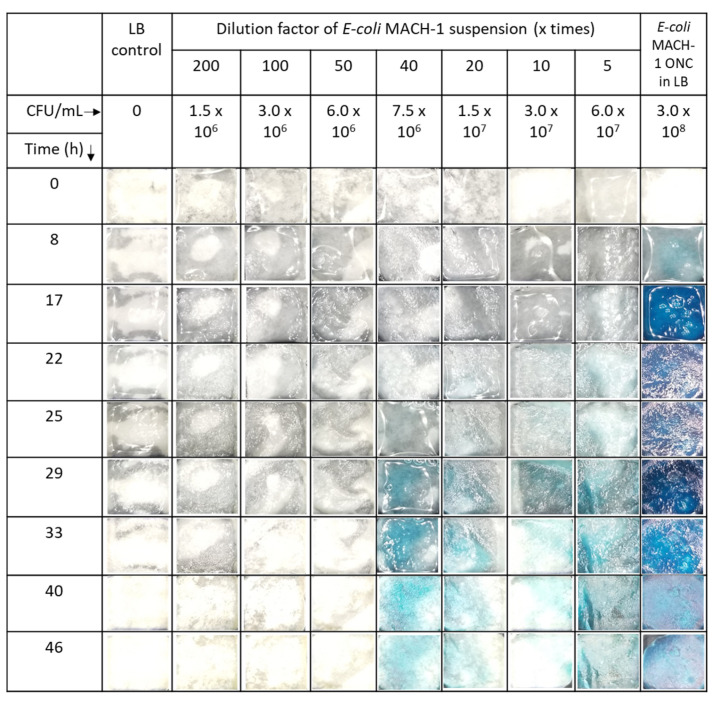
Photographs of visible color change of chitosan-g-X-Gluc coated paper after incubation in differently concentrated *E. coli* Mach1 suspensionsin LB.

Similarly, the paper sensors were exposed to 100 µL of a suspension of the nonpathogenic *E. coli* Mach1. The desired dilutions of bacteria suspension were prepared in LB, applied to the paper and analyzed by photography after designated reaction times at 20 °C (Figure 4). Depending on the dilution factor of the bacterial suspension and the reaction time, the characteristic blue color of the indigo dye was observed.

## 3. Discussion

The contact angle values (Figure S1) showed that the hydrophilic paper was coated with the much more hydrophobic chitosan. The successful deposition was also concluded from FESEM images (Figure S3) and was in agreement with the literature, where it was observed that the chitosan solution filled the micropores of uncoated paper and formed a layer on the surface [31]. The increasing values of the static water contact angle with increasing number of coating steps may indicate a thicker or less defective chitosan layer. Since paper with larger mean pore size exhibited larger values of the contact angle, the data are consistent with a chitosan covered cellulose paper that retained its microscopic structure. This interpretation is supported by morphological observations made by FESEM (Figure S3) and the analysis of the chemical composition of the surface by XPS (Figure 2a–c and Figure S2). As there was no nitrogen found in the paper used (Figure S2) and since XPS provides compositional information up to a maximum information depth of 8–10 nm under the conditions employed, one may conclude from the N1s signal observed only after coating with nitrogen containing chitosan (Figure 2) that the deposition was successful. Hence after coating the paper with chitosan, the characteristic N1s signal indicates the presence of chitosan in the top 8–10 nm of the sample surface. Furthermore, the element scan shown in Figure 2 corroborate that the chemical environment of the observed carbon and oxygen is consistent with chitosan. On the other hand, the not perfectly compensated charging make a quantitative analysis difficult.

The ATR-FTIR spectra (Figure 2d) were in agreement with previously published data [13], which showed that the chromogenic substrate X-Gluc was successfully immobilized on the chitosan layer coating of the filter paper. After modification with X-Gluc, the primary amine peak in chitosan chain at 1599 cm^−1^ disappeared, while the amide I and II vibrations at 1641 cm^−1^ and 1557 cm^−1^, respectively, became stronger.

The presence of the substrate residuals was also observed in enzymatic reaction under various conditions. Both in PBS and LB medium the neat enzyme solutions caused a characteristic blue color (Figure 3 and Figure S4), which did not leach from the paper. No blue color was observed outside hydrogel-coated paper, in agreement with previous report [17]. With increasing reaction time, the color became more intense. Qualitatively similar observation in *E. coli* suspension indicates the presence of the enzyme also in the LB medium (Figure 4), in agreement with previously published work [29]. The quantitative analysis of the photographs and the enzymatic reaction kinetics is key for an application of related sensors in the future. Bare eye visual detection possesses inherently lower LOD values [17] and is also hampered by possible observer bias as well as color weakness and blindness issues of the observer. The analysis mandates first a closer look at the fundamentals of the reaction kinetics involved.

### 3.1. Reaction Kinetics

The overall enzymatic reaction of substrate molecules in solution is classically described by the Michaelis–Menten kinetic scheme [32,33]:

(1) $$E+S\overset{K_{S}}{↔}\left(ES\right)\overset{k_{1}}{\to }P+E$$

Here *E* denotes the enzyme, *S* is the substrate and *P* is the primary reaction product, $k_{1}$ (in s^−1^) denotes the reaction rate constant, $K_{S}$ (in M) is the equilibrium constant of the dissociation of the enzyme-substrate complex (ES). A somewhat different substrate-conserving model is suggested for two phase reactions at solid substrates [34]:

(2) $$E+S\overset{K_{S}}{↔}\left(ES\right)\overset{k_{1}}{\to }P+S+E$$

Although this micro-kinetic scheme gives the same initial rate of product formation as that of Scheme (1), this reaction does not slow down at higher degrees of substrate conversion and finally yields infinite amounts of *P*. In addition, the scheme is oversimplified due to an irreversible catalytic second stage. In the present study this problem has been solved by a fast irreversible aerobic oxidation of the colorless *P* to the indigo-like dimer P, which is observed owing to its deep blue color

(3) $$2P+O_{2}=P+2H_{2}O$$

**Figure 5:**
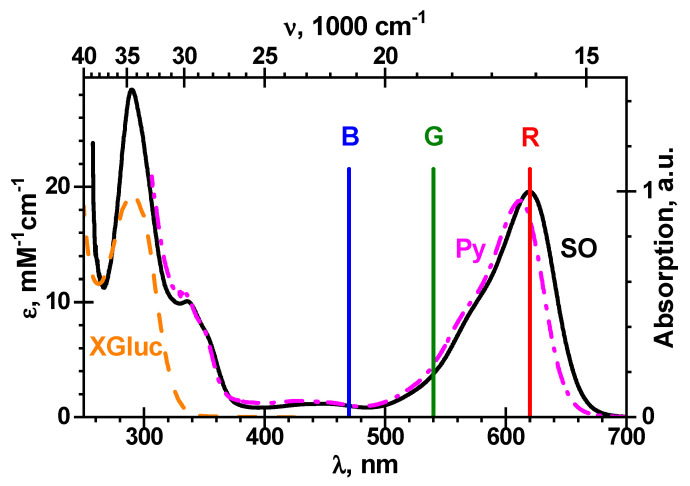
Absorption spectra of chromogenic substrate *S*, X-Gluc (X-Gluc, dash, orange; for chemical structure, see Figure 1, in PBS, extinction coefficient (ε) of indigo in dimethyl sulfoxide (DMSO) (SO, solid, black) and pyridine (Py, dash dot, magenta) at 25 °C. The maxima of indigo in DMSO are located as $\varepsilon_{max}$ = 19,600 and 28,500 M^−1^cm^−1^ at 619.6 and 289.0 nm, respectively, while the maximum of indigo in pyridine is found as $\varepsilon_{max}$ = 18,800 M^−1^cm^−1^ at 611.7 nm. The centers of red-green-blue (*RGB*) bands [35] are indicated with the vertical lines.

In the experiments, the enzymatic cleavage of chitosan-g-X-Gluc by ß-Gus leads to the primary product *P*, 5-bromo-4-chloro-3-hydroxyindole, and finally by reaction (3) to an insoluble final product P, 5,5’-dibromo-4,4’-dichloro-indigo, with a stoichiometric coefficient *b* = 1/2. The amount of reaction product at time *t* is estimated by a Samsung SM-G950W color digital image sensor having three primary red-green-blue (*RGB*) bands with maxima at 470, 540 and 620 nm, respectively [35]. The final reaction product, i.e., the blue pigment P, possesses strong absorption in the *R*-channel and practically no absorption both in the *G*-channel and in the *B*-channel, see Figure 5.

Hence, the *R*-channel is optimal for the detection of P. The experimental intensities $I_{RGB}$ were estimated as integral intensities in the *RGB*-channels, followed by the splitting of the measured images with ImageJ software. Because these *RGB* chromatic intensities are just reflected light intensities, they can be used for this purpose in a similar manner as a measure for P in the form of the Beer’s law. The amount of P is given by the relative color intensity at time *t* $\left(I_{R}\right)$ and that at time zero $(I_{R0})\;-\ln\frac{I_{R}}{I_{R0}}=aP$, where a>0 is an effective natural molar absorption coefficient of P over the red spectral range. Then for a given time *t* the pigment $P=-\frac{1}{a}\ln\frac{I_{R}}{I_{R0}}$ and amount of primary product formed in (1) equals $P=-\frac{1}{ab}\ln\frac{I_{R}}{I_{R0}}$. Taking into account that the value of $P=S_{0}-S,$ normalized to initial (*t* = 0) substrate amount $S_{0}$, the experimental value of the remaining substrate fraction, $x_{e}=\frac{S}{S_{0}}$, can be evaluated as:

(4) $$1-x_{e}=-\frac{1}{abS_{0}}\ln\frac{I_{R}}{I_{R0}}$$

Reaction scheme (1), similar to reference [36], gives for the total enzyme concentration $E_{0}$ the integrated enzyme kinetics:

(5) $$-\frac{K_{S}}{S_{0}}\ln x_{e}-x_{e}+1=\frac{k_{1}}{S_{0}}E_{0}t$$

### 3.2. Chromatic Intensity Kinetics at Different Enzyme Concentrations

**Figure 6:**
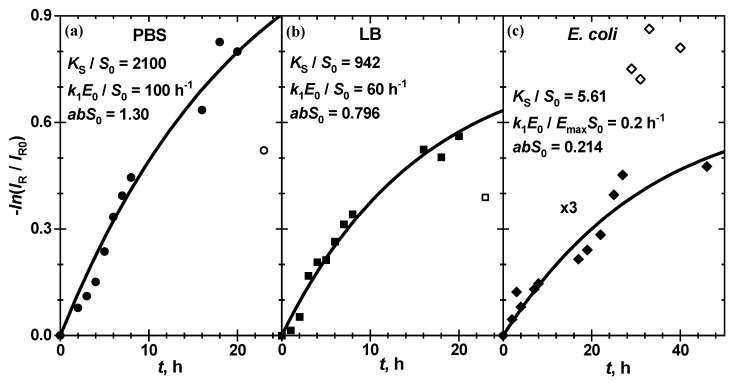
Evolution of the relative chromatic intensity $-\ln\frac{I_{R}}{I_{R0}}$ for the enzymatic cleavage of chitosan-g-X-Gluc by ß-Gus (**a**) in PBS at pH 7.4 (25 °C), (**b**) in LB at pH 7.0 (25 °C) as well as (**c**) in *E. coli* Mach1 bacterial suspension in LB at pH 7.0 (20 °C). The enzyme concentrations are 100 nM for (**a**) and 60 nM for (**b**). The stock bacterial suspension was diluted 5 times, corresponding to a bacteria concentration of 6 × 10^7^ colony-forming units (CFU)/mL. (**c**). The fit function (5) to the filled circles data points is shown with the solid line. The thermodynamic and kinetic constants as well as the scaling factor $abS_{0}$ are shown in the panels. The outlier data points, which are not included in the analysis, are shown as open symbols.

The typical kinetics of $-\ln\frac{I_{R}}{I_{R0}}$ for the enzymatic reaction of chitosan-g-X-Gluc with ß-Gus in PBS and in LB at 25 °C as well as in the presence of *E. coli* Mach1 strain in LB at 20 °C, respectively, is shown in Figure 6.

The chroma red exhibits a gradual decrease with reaction time for the chitosan film both in the enzymatic and in bacterial suspension solution, and clear tendency to level off at long reaction times. This is due to the absorption of red light by the blue pigment P, a product of reactions (1) and (3). The hydrolysis kinetics in all these three cases is well described by the Michaelis reaction scheme (1), as is shown by the solid line calculated with Equation (5) for all kinetic curves at different enzyme or bacterial suspension concentrations. Of course, the pigment P formation is slower at lower $E_{0}$ and at a higher dilution factor:

(6) $$d=\frac{E_{max}}{E_{0}}$$

where $E_{max}$ is the enzyme concentration in the stock bacterial suspension. The thermodynamic and kinetic parameters of (5), $k_{1}$ and $K_{S}$, are strongly correlated to each other. For almost equivalent fit quality, characterized by sum of squared residuals (SSR), several sets of kinetic and thermodynamic parameters $k_{1}$ and $K_{S}$ can be used, keeping the $\frac{k_{1}\;}{K_{S}}$ ratio practically constant. From the data in Table 1 one can see that even when these parameters change within at least one order of magnitude (cf. $\frac{K_{S}}{S_{0}}$ = 1000 and 10,000 for enzymatic substrate hydrolysis in PBS and LB as well as $\frac{k_{1}}{S_{0}}$ = 1 and 10 nM^−1^h^−1^ for bacterial substrate hydrolysis) the $\frac{k_{1}\;}{K_{S}}$ ratio changes only by 0.1%, 0.1% and 20%, respectively, so that the fit quality SSR remains practically constant within 0.06%.

**Figure 7:**
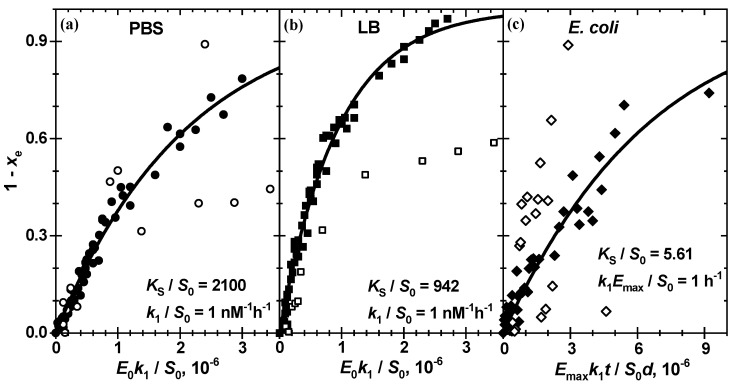
The kinetics of formation of primary product *P* for theenzymatic cleavage of chitosan-g-X-Gluc by ß-Gus (**a**) in PBS at pH 7.4 (25 °C), (**b**) in LB at pH 7.0 (25 °C) as well as (**c**) in *E. coli* Mach1 bacterial suspension in LB at pH 7.0 (20 °C). The experimental and theoretical x- and (**a**,**b**) $\frac{k_{1}}{S_{0}}E_{0}t$ - or (**c**) $\frac{k_{1}E_{max}}{S_{0}d}t$ -values are calculated according to Equations (4) and (5) with (**a**) $\frac{K_{S}}{S_{0}}$ = 2100, $\frac{k_{1}}{S_{0}}$ = 1 nM^−1^h^−1^, $E_{0}$ = 15, 30, 60, 100, 125 and 150 nM and $abS_{0}$ = 0.816, 0.555, 1.05, 1.30, 1.41, 1.34, (**b**) $\frac{K_{S}}{S_{0}}$ = 940, $\frac{k_{1}}{S_{0}}$ = 1 nM^−1^h^−1^, $E_{0}$ = 15, 30, 60, 100, 125 and 150 nM and $abS_{0}$ = 0.577, 1.09, 0.796, 1.08, 1.01 and 0.996 and (**c**) $\frac{K_{S}}{S_{0}}$ = 5.61, $\frac{k_{1}}{S_{0}}E_{max}$ = 1 h^−1^, bacterial suspension (3 × 10^8^ CFU/mL) dilution factor d = 5, 10, 20, 40, 80, 100 and 200 and $abS_{0}$ = 0.214, 0.174, 0.284, 0.843, 0.459, 0.255 and 0.793, respectively. The solid lines are calculated by fitting (5) through the filled data points at all different (**a**,**b**) $E_{0}$ and (**c**) d. The outlier data points, which are not included in analysis, are shown as open symbols.

While the thermodynamic equilibrium constant $K_{S}$ appears in the left side of (5), the kinetic rate constant $k_{1}$ and analytical enzyme concentration $E_{0}$ are present only on the right side as a product. In this case the roots $x_{e}$ of (5) *vs.* unitless time $\frac{k_{1}}{S_{0}}E_{0}t$ form a universal global line for all enzymatic kinetics in a series with different $E_{0}$ or d. The results of such a global fit for enzymatic reactions in PBS, LB and bacterial suspension shown in Figure 7 and Table 2 allow one to conclude that the enzymatic reaction of chitosan-g-X-Gluc in a complete set of analytical enzyme concentrations can be quantitatively described by the Michaelis–Menten reaction scheme (1) with only two generalized constants: (i) the thermodynamic equilibrium constant $\frac{K_{S}}{S_{0}}$ and (ii) the reaction rate constant $\frac{k_{1}\;}{S_{0}}$. In these figures, the experimental data $-\ln\frac{I_{R}}{I_{R0}}$ scaled with the factor $abS_{0}$ to an attained substrate conversion $1-\frac{S}{S_{0}}$ are plotted against the unitless time $\frac{k_{1}}{K_{S}}E_{0}t$ or $\frac{k_{1}E_{max}}{S_{0}d}t$. Taking the practically straightforward correlation of $\frac{K_{S}}{S_{0}}$ and $\frac{k_{1}\;}{S_{0}}$ into account, see Table 1, the latter is assumed to be equal to 1 nM^−1^h^−1^.

The constant value of $\frac{K_{S}}{S_{0}}$ for different $E_{0}$ implies a constant substrate amount $S_{0}$ at each $E_{0}$ in a series. This means in turn equal amounts of substrate, which are converted finally to the pigment P in equal amounts at the end of reaction, i.e., equal amplitude factors $abS_{0}$. Although variation of $abS_{0}$ is not so strong in series, some substantial deviations, such as 0.82 and 0.56, in comparison to 1.28 ± 0.16 for ß-Gus in PBS (Figure 7a), 0.58 in comparison to 1.00 ± 0.12 for ß-Gus in LB (Figure 7b) and 0.843, 0.459 and 0.793 in comparison to 0.232 ± 0.048 for *E. coli* Mach1 suspension in LB (Figure 7c) are probably caused by inhomogeneous light scattering in these samples.

While the constants $\frac{k_{1}}{S_{0}}$ and $\frac{K_{S}}{S_{0}}$ are a function of substrate amount, their ratio $\frac{k_{1}}{K_{S}}$ is independent from $S_{0}$ for enzymatic solutions and proportional to the enzyme concentration in stock bacterial suspension $\frac{k_{1}}{K_{S}}E_{max}$ for bacteria mediated cleavage. When the small temperature difference is neglected, the ratio of this value for stock (d = 1) suspension ($\frac{k_{1}}{K_{S}}E_{max}$, Table 2) to an averaged value of $\frac{k_{1}}{K_{S}}$ = 0.86 pM^−1^h^−1^ for the model system with the same substrate and enzyme in the same LB (Table 2) gives an estimation of $E_{max}$ for stock *E. coli* Mach1 suspension in LB.

(7) $$E_{max}=\frac{\frac{k_{1}}{K_{S}}E_{max}}{\frac{k_{1}}{K_{S}}}\;=\;210\;\mathrm{nM}$$

### 3.3. Limit of Detection (LOD)

With the results obtained, the minimum detectable pigment fraction $(LOD_{s}$) value can be determined from the correlation of experimental values of $1-x_{e}$ *vs*. calculated values of $1-x_{c}$ shown in Figure 7 by the following relation [37,38]:

(8) $$LOD_{S}=3\frac{\sigma }{\gamma }$$

Here σ is root-mean-square deviation of $1-x_{e}$ from $1-x_{c}$ and γ is the slope, which is ≈1. The calculated $LOD_{s}$ values are collected in Table 2. The minimal reaction conversion degree to smartphone-based detection of the enzymatic reaction is rather high, 0.089–0.25, probably due to substantial scattering in the experimental chromatic intensity.

The minimal reaction time ($LOD_{t}$) required to reach the conversion $LOD_{S}$ at a certain enzyme concentration according to Equation (5) is inversely proportional to enzyme concentration:

(9) $$LOD_{t}=\frac{LOD_{S}-\frac{K_{S}}{S_{0}}\ln\left(1-LOD_{S}\right)}{\frac{k_{1}}{S_{0}}E_{0}}$$

**Figure 8:**
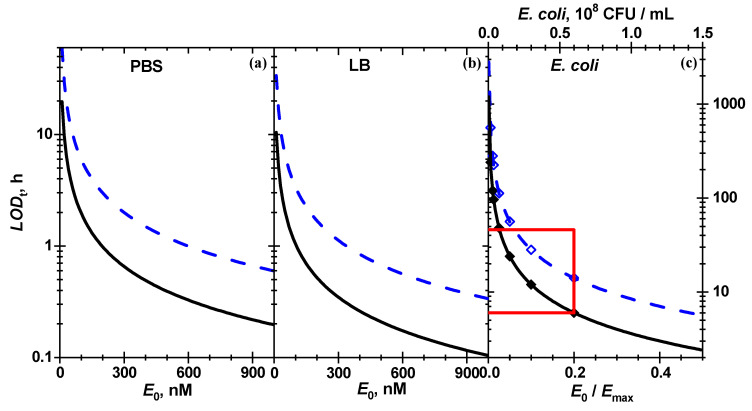
The concentration dependence of $LOD_{t}$ for the enzymatic cleavage of chitosan-g-X-Gluc by (**a**,**b**) β-Gus solutions and (**c**) *E. coli* Mach1 triggered enzymatic hydrolysis in bacterial suspension for the data included in the global fit (solid black) and for all data (dashed blue). (**c**) The bacterial concentration in the dispersions is shown on the top axis. The minimal reaction times for the dilution used in the experiment are shown with diamonds. The red rectangle limits the area of the *E. coli* Mach1 detection studied in the present work.

The lowest $LOD_{t}$ values in Table 2 calculated from the best fit from 100 to 440 nM h means that in order to detect β-Gus in LB at a low concentration of 1 nM, one needs 4–18 days to reach the minimal detectable pigment amount. The $LOD_{t}$ reaches a few (1–5) hours at 100 nM of enzyme. The concentration plot of $LOD_{t}$ in the entire range of studied $E_{0}$ is shown in Figure 8.

Optical biosensors that rely on the detection of absorbed ultraviolet–visible (UV–Vis) light [14,15,16,17,24,39,40,41,42] possess usually lower sensitivity than those based on fluorescence [13,16,18,19]. This is also true for UV–Vis detection of indigo derivatives [14,17,39]. Employing a Lowry protein assay, the absorption of a copper (I) complex at 660 nm measured by a smartphone camera enabled the detection of catalase with a rather high LOD of 5 µM [24]. Rarely the values of the LOD for photometric detection is lower than that for fluorometric detection [15]: LOD values of 20 and 45 nM were found for the hydrolysis of a chromogenic substrate by elastase and a fluorogenic substrate by α-glucosidase, respectively. This fact might be caused by different substrates used and variation of enzyme activity. In general, the LOD strongly depends on the quality of optical path and sample. Detection of β-Gus and β-galactosidase by formation of indigo-derivatives in transparent chitosan film was noted to possess LOD values of 3–5 nM for detection with an absorption spectrometer and around 30–60 nM for bare eye visual detection [17]. It was shown that with reporter-hydrogel coated glass 40 nM of β-Gus in solution can be detected within 5 min employing high-quality research-grade absorption spectrometers [14]. Here it is reported that for the same biochemical system, simple smartphone-based paper biosensors are shown to also reach this level, albeit with a 13 times longer incubation time of 1.6 h for the neat enzyme in PBS, which is comparable to the results reported before using a reporter-hydrogel coated glass fiber [14]. For neat enzyme in LB and *E. coli* bacterial suspension with a concentration of 5.8 × 10^7^ CFU/mL the incubation time for the smart phone approach required longer incubation time of 5–6 h (Table 2).

These data compared favorably to literature data. In an attempt to distinguish among *E. coli* serotypes exhibiting different phenotypes regarding production of the enzyme β-Gus, a multilayered paper sensor with two different indigo pigments duplex coloration was proposed by Kim et al. [39]. Without an additional cultivation step of *E. coli* but extra centrifugation and bacteria lysis steps, an LOD of ~10^7^ CFU/mL was found by colorimetric analysis with a commercial scanner or bare eye visual detection. A paper-based colorimetric β-lactamase biosensor that relies on the cleavage of nitrocefin showed a LOD of 3.8 × 10^6^ CFU/mL for *E. coli*, employing an absorption spectrophothometer [41]. While smartphone detection by absorption of 4-nitrophenol on paper exhibited low values of the LOD of 2, 13 and 100 nM for β-glucosidase, β-galactosidase and β-Gus, respectively, the triggering bacteria *Enterococcus faecalis* was found only at a high concentration of 1 × 10^9^ CFU/mL without pre-enrichment via culturing [42]. Thus, the simple sensor system presented here is comparable regarding its LOD values to other sensoric systems based on enzyme chromogenic substrates that are under development, but does not require any extra lysis step or sensitive reagents.

Nevertheless our POC bacterial detection approach needs to be further optimized for specific application to meet the sensitivity required to indicate the relevant harmful concentrations thresholds for *E. coli*, as used here as a model system, or other bacteria. Therefore, further developments towards applications should be focused on suppressing unwanted reflections, increasing the sensitivity by increasing the specific surface area e.g., by nanostructuring and by increasing the substrate loading as well as by implementing added features. This may refer to the integration of a bacteria lysis step and/or concentration of bacteria or target enzymes for instance via an initial filtration step. We emphasize that this work has to be classified as a proof of concept for chromogenic reporter hydrogel based *E. coli* detection integrated in a POC approach compatible smart phone signal read out that might be used in the long term upon further development as a POC test for bacterial contamination.

## 4. Conclusions

We conclude that the heterogeneous β-Gus catalyzed hydrolysis of the chromogenic substrate chitosan-g-X-Gluc can be quantitatively described within the Michaelis enzymatic reaction scheme. The ratios of reaction rate and equilibrium constants of the enzyme-substrate complex are about 0.3 and 0.9 pM^−1^h^−1^ for β-Gus in PBS and LB, respectively. The minimal substrate conversion degree and the minimal time required for detection of the indigo pigment in smartphone-based detection during the enzymatic reaction is about 0.15 and about 2 h at an enzyme concentration of 100 nM, respectively. Moreover we could show with the herein firstly described alterative analysis approach for reaction kinetics of chromogenic enzyme substrates that *E. coli* suspensions with concentrations of 5.8 × 10^7^ CFU/mL can be detected within 6.2 h under the conditions used with enzyme responsive hydrogel coated paper documented via a smart phone camera. The kinetic analysis yields accurate information on the reaction initial rate by taking into account the much more reliable signal at higher substrate conversion to determine the initial slope of the Michaelis–Menten kinetics with higher precision and sensitivity than the usual analysis of the initial rates. Therefore, this approach based on enzymatic substrate conversion via bacterial enzymes offers the potential to be used for quantitative, low cost smart phone supported POC detection systems that can be tailored and improved for various settings, where sensitive enzyme targeting from bacteria sources or others is needed.

## 5. Materials and Methods

### 5.1. Materials

Chitosan (medium molar mass, 190–310 kDa, 75–85% deacetylated), phosphate buffered saline (PBS) tablets, 5-bromo-4-chloro-3-indolyl-β-D-glucuronide (X-Gluc), *N*-(3-dimethylaminopropyl)-*N*-ethylcarbodiimide hydrochloride (EDC·HCl), *N*-hydroxy succinimide (NHS), β-glucuronidase (ß-Gus) purified from *E. coli* (694.3 units/mg, E.C. 3.2.1.31; type IX-A), dimethyl sulfoxide (DMSO, 99%), pyridine (anhydrous, 99.8%) were purchased from Sigma-Aldrich. Indigo (Carl Roth) and acetic acid (glacial, J. T. Baker), Dulbecco’s phosphate saline buffer (DPBS, 10 ×, 95 mM (PO_4_) without Mg^2+^ and Ca^2+^, Lonza), Lysogeny broth (LB, Luria/Miller: 10 g/L tryptone, 5 g/L yeast extract, 10 g/L NaCl, pH 7.0 ± 0.2, Carl Roth GmbH, Germany), qualitative filter paper (5–12 µm and 12–15 µm pore size, VWR, France) were purchased from the listed suppliers. Milli-Q water was drawn from a Millipore Direct Q8 system with a resistivity 18.2 MΩ cm (Millipore advantage A10 system, Schwalbach, with Millimark Express 40 filter, Merck, Germany), sterilized PBS solution (10 mM, pH 7.4) was prepared by dissolving one tablet of phosphate buffered saline in 200 mL of Milli-Q water followed by autoclaving at 121 °C and 100.1 kPa vapor pressure in an autoclave (Systec VB-150, Systec GmbH, Linden, Germany). Only for the data shown in Figure S4, PBS was obtained through the dilution of DPBS with Milli-Q water and volume ratio of DPBS and Milli-Q water was 1:9.

### 5.2. Bacteria

As a test organism we used the nonpathogenic *Escherichia coli* W (ATCC9637) derivative Mach1™ (T1 Phage-resistant, chemical competent, purchased form Invitrogen, USA) [43].

### 5.3. Preparation of Chitosan-Coated Paper 

100 µL of 1.5 wt% chitosan in 1 wt% acetic acid solution was deposited on paper substrates (0.8 × 1.0 cm^2^) and dried these overnight. The two-time deposition of chitosan was done by adding 100 µL of chitosan solution on dried pre-deposited paper substrate followed by overnight drying in a flow hood. 

### 5.4. Contact Angle Measurements

The contact angle measurements were taken using a Data Physics Instruments (Filderstadt, Germany) Contact Angle System OCA-15, equipped with a video measuring system with high-resolution charged coupled device (CCD) camera. SCA 20 software was used for data acquisition. Chitosan coated papers were fixed on a glass slide and were kept flat throughout the analysis. The static contact angle was measured by the sessile drop method by gently placing a droplet of 2 μL of Milli-Q water, and the values were collected 5 times.

### 5.5. Attenuated Total Internal Reflection-Fourier Transform Infared (ATR-FTIR) Spectroscopy

A Tensor 27 Bruker Optik GmbH (Ettlingen, Germany) FTIR spectrometer was used for ATR-FTIR analysis. The chitosan coated paper was fixed on a holder and subsequently placed in the chamber of the instrument. The measurements were performed in absorbance mode in the spectral range 4000 to 400 cm^−1^ and a spectral resolution of 4 cm^−1^. The background spectra were obtained by using air.

### 5.6. Field-Emission Scanning Electron Microscopy (FESEM) Analysis

The dried samples were sputtered with a thin gold layer (8–10 nm) in a sputter coater (S150B BOC Edwards, West Sussex, UK) at a pressure of 0.2 mbar in argon atmosphere for 2 min at a voltage of 1.0 kV and then placed in a sample holder in the FESEM sample chamber. The data were acquired on a FESEM (Zeiss Ultra 55cv, Oberkochen, Germany) with 30 kV maximum operating voltage formed with an operating voltage of 10 kV with the in-lens secondary electron detector.

### 5.7. X-ray Photoelectron Spectroscopy (XPS) Analysis

The surface chemical composition of the films was analyzed by X-ray photoelectron spectroscopy (S-probe ESCA SSX-100s, Surface Science Instruments, Mountain View, USA) with an Al Kα radiation of 200 W. Survey spectra were measured from 0 to 1200 eV with a resolution of 1 eV (spot size: 800 µm^2^) and high resolution spectra with a resolution of 0.1 eV (spot size: 300 µm^2^).

### 5.8. Modification with 5-Bromo-4-Chloro-3-Indole-β-D-Glucuronide Sodium Salt (X-Gluc)

Chitosan-g-X-Gluc paper substrate was prepared using EDC/NHS chemistry according to the reference [16]. Briefly, 4.5 mM of X-Gluc was prepared in PBS (pH 7.4), followed by addition of EDC (6.7 mol/mol of X-Gluc) and NHS (6.7 mol/mol of X-Gluc). Then the solution was stirred for 1 h. Each chitosan sample was then immersed in 2 mL of this modification solution for 6h under shaking (rate 60 Hz), followed by washing with Milli-Q water for 2 h under shaking, with subsequent exchange of Milli-Q water after each 30 min. Then the samples were dried overnight in a flow hood.

### 5.9. Enzymatic Reaction with β-Glucuronidase

20 µM stock enzyme solution of ß-Gus was prepared in sterilized PBS (pH 7.4) and the desired enzyme concentrations were prepared by further dilutions in PBS (pH 7.4) and LB (pH 7.0). 100 µL of the enzyme solutions tested were deposited on the paper sensors and incubated at 25 °C. Pictures were taken at different time intervals with a Samsung Galaxy S8 camera in a standardized position on a black box with integrated illumination, designed and produced by the workshop of the University of Siegen (see Figure S5). The illumination was done with a LIVARNO LUX light-emitting diode (LED) light strip (100–240 V, 50/60 Hz) fixed within the black box. 

### 5.10. Detection of E. coli Cultures

For studying enzymatic cleavage via ß-Gus produced by *E. coli* Mach1, overnight culture was prepared by taking 1 colony from an agar plate prepared in 5 mL LB and incubation at 37 °C for 18 h with shaking at 200 rpm. The OD600 nm of overnight culture was set to 0.5 referring to a bacteria concentration of (3 ± 0.2) × 10^8^ CFU/mL. Then the desired dilutions of bacteria suspension were prepared in LB. We deposited 100 µL of each dilution of bacteria solution on the paper sensors for the experiment. All the experimental steps were performed at 20 °C.

## Figures and Tables

**Table 1:** Correlation of parameters $K_{S}$ and $k_{1}$ in Equation (5) for the enzymatic reaction of chitosan-g-X-Gluc with ß-Gus in PBS at pH 7.4 (25 °C) and in LB at pH 7.0 (25 °C) as well as in *E. coli* Mach1 bacterial suspension in LB at pH 7.0 (20 °C).

Experiment	$E_{0},\;\mathrm{nM}$	Set	$\frac{K_{S}}{S_{0}}$	$\frac{k_{1}}{S_{0}},\;{\mathrm{nM}}^{-1}h^{-1}$	${\mathrm{abS}}_{0}$	$\frac{k_{1}}{K_{S}},\;{\mathrm{pM}}^{-1}h^{-1}$	1000 × SSR ^a^
Enzyme/PBS	100	1	2100	1 ^b^	1.30	0.475	28.2
Enzyme/PBS	100	2	1000 ^b^	0.476	1.30	0.476	28.2
Enzyme/PBS	100	3	10,000 ^b^	4.75	1.30	0.475	28.2
Enzyme/LB	60	1	942	1 ^b^	0.796	1.06	8.13
Enzyme/LB	60	2	1000 ^b^	1.06	0.796	1.06	8.13
Enzyme/LB	60	3	10,000 ^b^	10.6	0.796	1.06	8.13
Bacteria/LB	5 ^c^	1	5.61	1 ^b,d^	0.214	0.178 ^e^	2.38
Bacteria/LB	5 ^c^	2	10 ^b^	1.62 ^d^	0.221	0.162 ^e^	2.38
Bacteria/LB	5 ^c^	3	68.7	10 ^b,d^	0.228	0.146 ^e^	2.39

^a^ Sum of squared residuals. ^b^ Fixed parameter in optimization. ^c^ Value d (6). ^d^$\;\frac{k_{1}}{S_{0}}E_{max}$ in h^−1^. ^e^ $\frac{k_{1}}{K_{S}}E_{max}$ in h^−1^.

**Table 2:** Global fit of the hydrolysis kinetics of chitosan-g-X-Gluc by ß-Gus in PBS at pH 7.4 (25 °C), in LB at pH 7.0 (25 °C) as well as in *E. coli* Mach1 suspension in LB at pH 7.0 (20 °C).

Experiment	*E*_0_^a^, nM	$\frac{K_{S}}{S_{0}}\;$	$\frac{k_{1}}{S_{0}}$^b^, nM^−1^h^−1^	$\frac{k_{1}}{K_{S}}$, pM^−1^h^−1^	*LOD_S_* ^c^	*LOD_t_*^c^, nM h
Enzyme/PBS 1	15–150	2100	1	0.475	0.089 (0.25)	200 (600)
Enzyme/PBS 2	30–500	7760	1	0.129	0.099 (0.20)	790 (1700)
Enzyme/LB 1	15–150	942	1	1.06	0.10 (0.30)	100 (340)
Enzyme/LB 2	30–500	1540	1	0.650	0.25 (0.43)	440 (860)
Bacteria/LB	5–200 ^d^	5.61	1 ^e^	0.178 ^f^	0.16 (0.34)	1.2 (2.8) ^g^
	210 ^h^					250 (590)

^a^ Range of enzyme concentrations. ^b^ Fixed parameter in optimization. ^c^ For data in the global fit (all data). ^d^ Value d (6). ^e^ $\frac{k_{1}}{S_{0}}E_{max}$ in h^−1^. ^f^ $\frac{k_{1}}{K_{S}}E_{max}$ in h^−1^. ^g^ In h. ^h^ $E_{max}$ estimated with Equation (7).

## Data Availability

The data presented in this study are available on request from the corresponding author. The data are not publicly available as they are part of an on-going study.

## Supplementary Materials

The following are available online at https://www.mdpi.com/2079-6374/11/1/25/s1, Figure S1. Water contact angle data for chitosan solutions with different concentrations deposited 1 time and 2 times on (**a**) 5–12 µm pore-sized and (**b**) on 12–15 µm pore-sized paper substrates; Figure S2. XPS survey scan of chitosan-modified paper (**a**) one and (**b**) two depositions from 1.5 wt% chitosan solution in acetic acid) as well as bare non-modified paper (**c**) and (**d**) on different sides of the paper); Figure S3. Field-emission scanning electron microscopy (FESEM) images of sensor paper: (**a**) uncoated paper and (**b**) paper coated with 1 wt%, (**c**) 1.5 wt% and (**d**) 2 wt% chitosan solution, 5–12 µm; Figure S4. Photographs of sensor hydrogel coated paper after reaction with enzyme: (**a**) uncoated paper control, (**b**) X-Gluc functionalized paper (one-time chitosan deposition) tested with bacteria suspension with bacteria concentration of 1.3 × $10^{7}$ CFU/mL, (**c**) X-Gluc functionalized paper (two times chitosan deposition) tested with bacteria suspension with bacteria concentration of 1.3 × $10^{7}$ CFU/mL, (**d**) X-Gluc functionalized paper (two-times chitosan deposition) tested with in neat enzyme solution (1 µM) phosphate-buffered saline (PBS). The incubation time was in all cases 4 h; Figure S5. Schematic of the black box design. The box possesses (1) a camera hole and (2) a platform to hold the smart phone, a water-reservoir chamber and a sample holder, where (3) paper supported samples of a diameter of 8 mm fit in and can be easily detached for cleaning in case of contamination. The box was designed and drawn using inventor drawing software; Table S1. Binding energies and the atomic concentration of C1s, O1s and N1s for one-time deposition and two-times deposition; Table S2. Assignment of most prominent bands observed in ATR-FTIR spectra for chitosan films on paper (2 coatings, 1.5 wt%) and chitosan on paper (2 coatings, 1.5 wt%) grafted with chromogenic substrate X-Gluc.
