# Supplementary material for: Quantitative E. coli Enzyme Detection in Reporter Hydrogel-Coated Paper Using a Smartphone Camera
Source: Biosensors (Basel). 2021 Jan 19;11(1):25. doi: 10.3390/bios11010025 (PMC7833396; doi:10.3390/bios11010025)
Supplement: Supplementary file 1 [file biosensors-11-00025-s001.pdf]

Supplementary Materials

Article

Quantitative *E. Coli* Enzyme Detection in Reporter Hydrogel-Coated Paper Using a Smartphone Camera

Kawaljit Kaur [1,†], Winny Chelangat [1,2,†], Sergey I. Druzhinin [1,*], Nancy Wangechi Karuri [3], Mareike Müller [1] and Holger Schönherr [1,*]

[1] Physical Chemistry I & Research Center of Micro and Nanochemistry and Engineering (*Cμ*), Department of Chemistry and Biology, University of Siegen, Adolf-Reichwein-Straße 2, 57076 Siegen, Germany; Kawaljit.Kaur@uni-siegen.de (K.K.); chelangat.winny16@students.dkut.ac.ke (W.C.); m.mueller@chemie-bio.uni-siegen.de (M.M.)

[2] Mechatronics Engineering Department, Dedan Kimathi University of Technology, Nyeri-Mweiga Road, 10143 Nyeri, Kenya

[3] Chemical Engineering Department, Dedan Kimathi University of Technology, Nyeri-Mweiga Road, 10143 Nyeri, Kenya; nancy.karuri@dkut.ac.ke

† These authors contributed equally to this work.

* Correspondence: druzhinin@chemie.uni-siegen.de (S.I.D.); schoenherr@chemie.uni-siegen.de (H.S.)

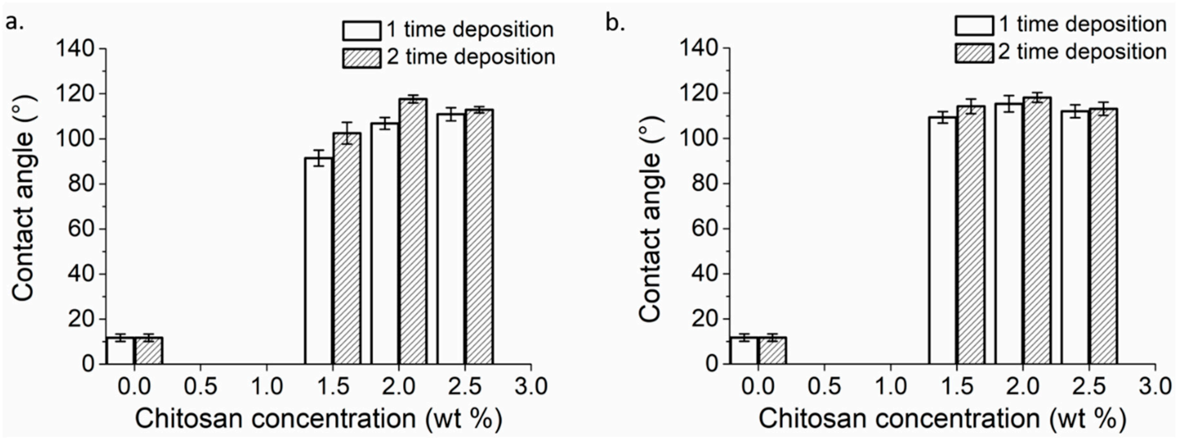

Figure S1. Water contact angle data for chitosan solutions with different concentrations deposited 1 time and 2 times on (**a**) 5–12 μm pore-sized and (**b**) on 12–15 μm pore-sized paper substrates.

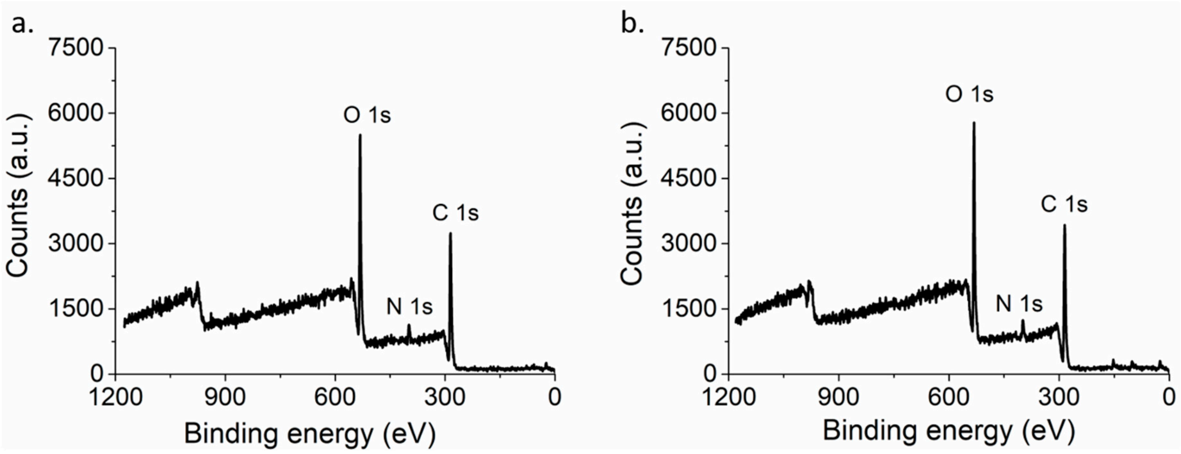

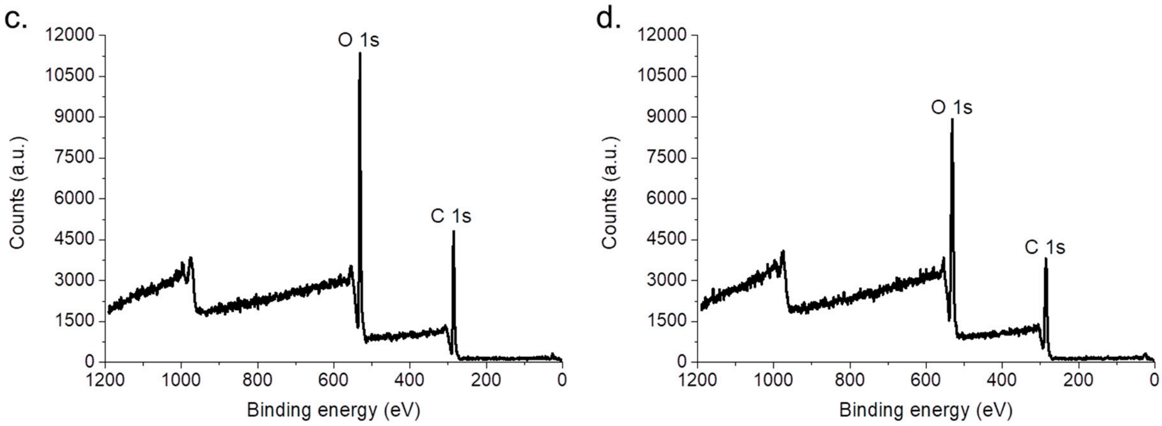

Figure S2. XPS survey scan of chitosan-modified paper (**a**) one and (**b**) two depositions from 1.5 wt% chitosan solution in acetic acid) as well as bare non-modified paper (**c**) and (**d**) on different sides of the paper).

Table S1. Binding energies and the atomic concentration of C1s, O1s and N1s for one-time deposition and two-times deposition.

Name	Binding Energies Position (eV)		Atomic Concentration (atom-%)	
	One time Deposition	Two Times Deposition	One time Deposition	Two times Deposition
C1s	284.9	285.6	63.6	63.1
O1s	531.9	533.1	31.0	33.0
N1s	398.9	400.2	3.4	3.8

Table S2. Assignment of most prominent bands observed in ATR-FTIR spectra for chitosan films on paper (2 coatings, 1.5 wt %) and chitosan on paper (2 coatings, 1.5 wt %) grafted with chromogenic substrate X-Gluc.

Vibrations and Assignments	Chitosan		Chitosan Grafted X-Gluc
	Data	Refs S1,S2	
ν_s (O-H, N-H)	3334	3435–3330	3335
ν (C-H) $_{aromatic}$	-	-	3074
ν (C = O) amide I	1645	1620–1655	1641
δ_b (NH_2)	1599	1590–1610	-
ν (N-H) amide II	1553	1550–1565	1557
δ_b (C-H)	1375	1375–1382	1376
ν_s (C-O-C)	1053	1065–993	1065
ν_s (C-O-C bridge)	896	890–900	898

S1. Kasaai, M. R. A Review of Several Reported Procedures to Determine the Degree of *N*-Acetylation for Chitin and Chitosan Using Infrared Spectroscopy. *Carbohydr. Polym.* **2008**, *71*, 497-508, DOI: 10.1016/j.carbpol.2007.07.009.

S2. Rahman, N. A.; Abu Hanifah, S.; Mobarak, N. N.; Su'ait, M. S.; Ahmad, A.; Shyuan, L. K.; Khoon, L. T. Synthesis and Characterizations of o-Nitrochitosan based Biopolymer Electrolyte for Electrochemical Devices. *PLOS ONE* **2019**, *14*, e0212066, DOI: 10.1371/journal.pone.0212066.

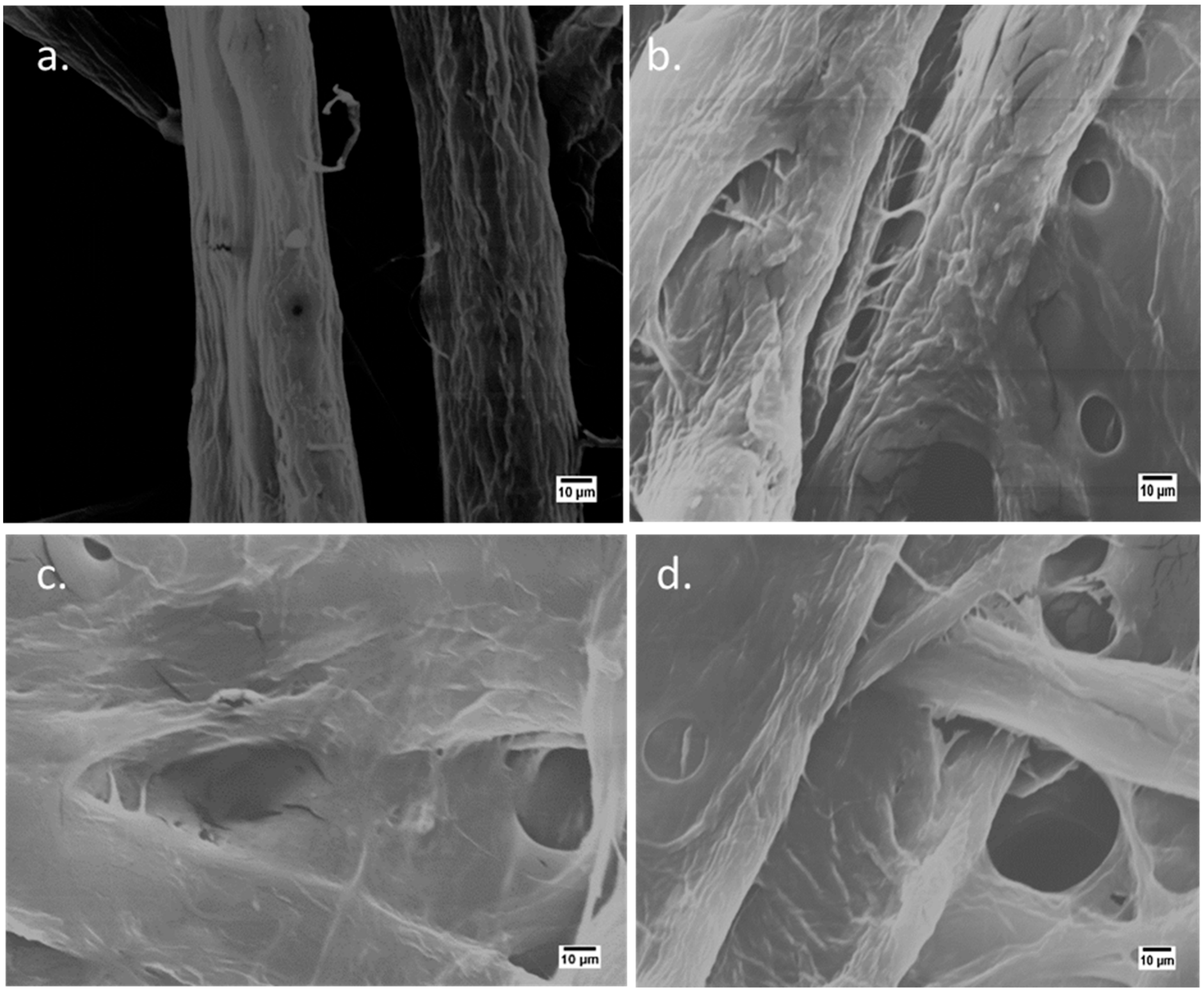

Figure S3. Field-emission scanning electron microscopy (FESEM) images of sensor paper: (**a**) uncoated paper and (**b**) paper coated with 1 wt%, (**c**) 1.5 wt% and (**d**) 2 wt% chitosan solution, 5–12 µm.

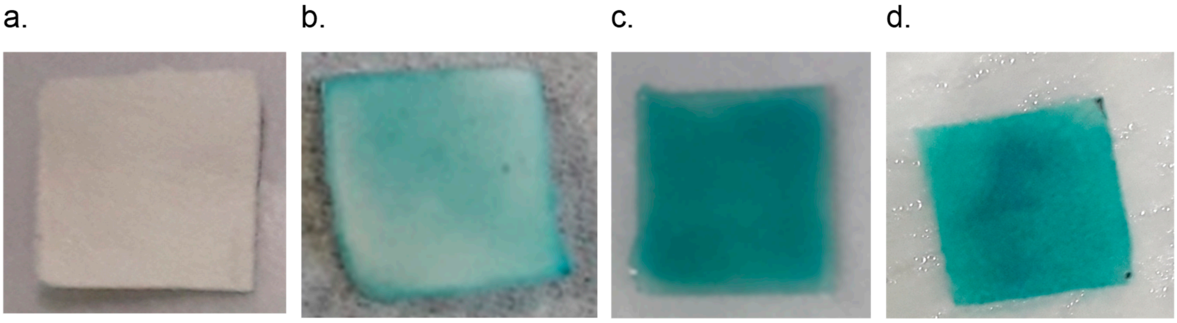

Figure S4. Photographs of sensor hydrogel coated paper after reaction with enzyme: (**a**) uncoated paper control, (**b**) X-Gluc functionalized paper (one-time chitosan deposition) tested with bacteria suspension with bacteria concentration of 1.3 × 10^7CFU/mL, (**c**) X-Gluc functionalized paper (two times chitosan deposition) tested with bacteria suspension with bacteria concentration of 1.3 × 10^7 CFU/mL, (**d**) X-Gluc functionalized paper (two-times chitosan deposition) tested with in neat enzyme solution (1 µM) phosphate-buffered saline (PBS) prepared by dilution of DPBS. The incubation time was in all cases 4 h.

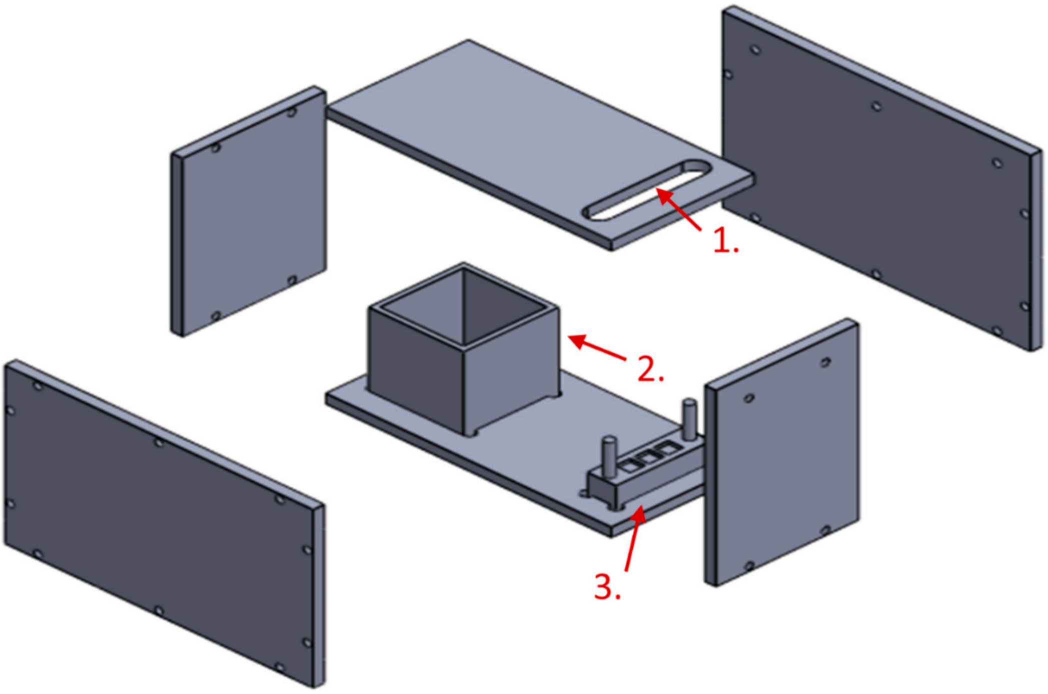

Figure S5. Schematic of the black box design. The box possesses (1) a camera hole and (2) a platform to hold the smart phone, a water-reservoir chamber and a sample holder, where (3) paper supported samples of a diameter of 8 mm fit in and can be easily detached for cleaning in case of contamination. The box was designed and drawn using inventor drawing software.
